# Investigation of Double-Band Electrophoretic Pattern of ITS-rDNA Region in Iranian Isolates of *Leishmania tropica*

**Published:** 2013

**Authors:** MA Ghatee, I Sharifi, H Mirhendi, Z Kanannejad, G Hatam

**Affiliations:** 1 Dept. of Parasitology and Mycology, School of Medicine, Kerman University of Medical Sciences, Kerman, Iran; 2 Leishmaniasis Research Center, Kerman University of Medical Sciences, Kerman, Iran; 3 Dept. of Parasitology and Mycology, School of Public Health, National Institute of Health Research, Tehran University of Medical Sciences, Tehran, Iran; 4 Dept. of Immunology, School of Medicine, Shiraz University of Medical Sciences, Shiraz, Iran; 5 Dept. of Parasitology and Mycology, School of Medicine, Shiraz University of Medical Sciences, Shiraz, Iran

**Keywords:** *Leishmania tropica*, Double-band, ITS, Iran

## Abstract

**Background:**

*Leishmania tropica* is a genetically divergent species. Amplification of entire internal transcribed spacer (ITS) region of *L. tropica* isolates obtained from Bam district, one of the well known focus of anthroponotic cutaneous leishmaniasis (ACL) in Iran, revealed a double-band pattern in agarose gel electrophoresis. This study explains how this pattern occurs.

**Methods:**

Twenty seven *L. tropica* smear preparations were collected from Bam district, south east Iran, and eight *L. major* and one *L. infantum* smear preparations were gathered from Shiraz, south west Iran. Furthermore one *L. major* and one *L. infantum* cultured standard strains were tested using entire ITS-PCR to survey their electrophoretic pattern. The ITS sequences of *L. tropica*, *L. major*, and *L. infantum* already deposited in GenBank were analyzed. Analysis of GenBank sequences of *L. tropica* revealed two groups of sequences based on length size, one group having a 100 bp gap. Therefore, a new reverse primer namely LITS-MG was designed to exclude this gap in PCR products.

**Results:**

Whole ITS fragment amplification resulted in a double-band pattern in all *L. tropica* cases, while a sharp single band was observed for *L. infantum* and *L. major* isolates. This result was corresponding to the result obtained from *in silico* analysis of GenBank sequences. Use of LITS-MG primer was expectedly resulted in a single band including ITS1, 5.8s and partial ITS2 product for *L. tropica* which is appropriate for following molecular studies such as sequencing or restriction analysis.

**Conclusion:**

Sequences analysis of GenBank *L. tropica* sequences and following practical laboratory tests revealed at least two alleles in *L. tropica* which were confirmed in Bam isolates. This especial double-band pattern is because of a 100 bp fragment difference within ITS-rDNA alleles.

## Introduction

Leishmaniasis can cause a spectrum of manifestations due to the involvement of different parts of the body ranging from skin to spleen, resulting in self-improving infections to disabilities and death. Among more than 20 different species and subspecies of *Leishmania* genus, some are causative agents of several forms of leishmaniasis and others may induce special clinical forms of the disease (1). Cutaneous leishmaniasis (CL) has an annual incidence of 0.7-1.2 million cases in both Old and New Worlds (2). *Leishmania major*, *L. tropica*, *L. aethiopica*, *L. mexicana*, *L. amazonensis*, *L. panamensis*, *L. pruviana*, *L. guyanensis*, and *L. braziliensis* can cause leishmaniasis from self-healing cutaneous lesions to mucocutaneous forms (3). Nearly 90% of CL cases are reported from Afghanistan, Brazil, Peru, Saudi Arabia, Syria, and Iran (1). *L. tropica* is an ordinary causative agent of urban CL with a dry clinical form whose lesion may last up to three years (4) with approximately 30 zymodems and is appeared to be more polymorphic among the Old World *Leishmania* species (5). *L. tropica* is distributed in North Africa (Morocco and Tunisia), and new foci in south Sahara (Kenya and Namibia) to Middle East (Syria, Iran, Iraq, Saudi Arabia, Yemen, and Turkmenistan). The east border is restricted to Punjab and Rajasthan as north western states of India (4).

There are different methods and markers for discriminating *Leishmania* species or strains as well as determining their distribution in diverse geographical zones. PCR-based techniques are able to detect parasite in the culture or directly from the clinical samples with a high sensitivity and specificity (6, 7). Kinetoplastid DNA (kDNA)-PCR followed by restriction fragment length polymorphism (RFLP) (8, 9), ITS sequence analysis (10, 11), random amplification of polymorphic DNA (RAPD) (12), amplified fragment length polymorphism (AFLP) (13), and multilocus microsatellites typing (14, 15) have been used for genotyping of *Leishmania*. Different studies have applied ITS region of ribosomal DNA in order to survey the phylogenetic relationship and differentiation of *Leishmania* species and strains (6, 10, 11, 16–19).In a study (unpublished data), we assessed the heterogeneity of *L. tropica* strains isolated from Bam, a well-known anthroponotic cutaneous leishmaniasis (ACL) focus (20, 21) in Kerman province, south east Iran. We amplified the ITS region of ribosomal DNA (rDNA) by the already described primers, LITS V and LITS R (6). Electrophoretic pattern revealed a double-band. Interestingly some other researchers have also reported this double-band pattern of *L. tropica* in whole ITS (ref. 11; H. Hajjaran and A. Akhavan, personal communications).

The present study aims to explain how this two-band pattern occurs. The results of the study may help researchers to elucidate the delicate angles of molecular epidemiology of leishmaniasis.

## Materials and Methods

### Parasite and study area

Forty stained smear preparations from clinical samples were collected from typical CL patients who were referred to the Bam Cutaneous Leishmaniasis Control Center. Furthermore, 16 samples from patients suspected to be infected with *L. major* were obtained from Shiraz, center of Fars province, southwest Iran. According to microscopy examination, 27 out of 40 samples from Bam and 13 out of 16 samples from Shiraz were finally selected for following molecular survey. One bone marrow aspirate loaded with leishman bodies was also taken from a patient in Shiraz Shahid Faghihi Hospital. Moreover, one reference strain of *L. major* (MRHO/IR/75/ER) and a previously defined *L. infantum* (MCAN/IR/07/ Moheb-gh) preserved in RPMI 1640 culture media were kindly provided by Prof. Gholamreza Hatam (5^th^ author) from Shiraz University of Medical Sciences, Shiraz, Iran.

### DNA extraction

Skin tissue on smears was scratched off and collected in 1.5 ml microtubes containing lysis buffer (Tris 100mM, EDTA 10mM, NaCl 100mM. SDS 1%, Triton X 100 2%) and 10 µg /ml proteinase K was added and incubated at 56°C for one hour. The samples were extracted once with phenol/chloroform (25:24 v/v) and subsequently extracted once with chloroform. DNA was precipitated with equal volumes of isopropanol, washed with 70% ethanol, dried, and suspended in 50 µl ultrapure water and stored at -20 °C till use.

### PCR

For species identification, kDNA of all the samples were amplified by the primers 13Z (5’- ACT GGG GGT TGG GTG TAA AAT AG-3’) and LiR (5’- TCG CAG AAC GCC CCT -3’) (22) for species identification. The PCR mixture consisted of 12.5 µl of 2x premix (Amplicon, Denmark), 20 pmol of each primer, 5 µl of template DNA, and enough water up to 25µl reaction. The cycling PCR conditions was 95°C for 5 min followed by 35 cycles of 94°C for 45s, 55°C for 60s, and 72°C for 90s and then final extension at 72°C for 7 min in an Applied Biosystem thermocycler. PCR products were subjected to 1.2% agarose gel electrophoresis with 0.5 µg/ml ethidium bromides for 90 min at 80v in 1X TBE buffer (90mM Tris-HCL, 90 mM Boric Acid, 2 mM EDTA) and visualized by transiluminator. A 100 bp DNA size marker was used in each run.

The entire ITS1-5.8S-ITS2 in rDNA was amplified by LITSV (5- ACA CTC AGG TCT GTA AAC -3) and LITSR (5- CTG GAT CAT TTT CCG ATG -3) primers (6). The cycling program was 95°C for 5 min followed by 94°C for 45s, 55°C for 60s, and 72°C for 90s which were programmed for 35 cycles. Amplification program was terminated by a final extension at 72°C for 7 min. The products were electrophoresed as mentioned above. Negative controls (water instead of template DNA) were included in all PCR runs.

### Sequence analysis and design of new primer and PCR conditions

ITS-sequences of *L. tropica*, *L. major*, and *L. infantum* already deposited in GenBank were analyzed by Geneious Basic 5.5.6 software (23) and DNASIS MAX version 3.0 trial software (Hitachi Software Engineering Co., LTD.) and new reverse primer, LITS-MG (5- ATG GCC AAC GCG AAG TTG -3), was designed in order to exclude the additional fragment in the sequence. Besides, LITSR and LITS-MG primers were used in order to amplify a DNA sequence (namely ITSMG), including ITS1, 5.8S rRNA, and a part of the ITS2 sequence. The cycling programs were followed by a primary 95°C for 300s followed by 35 cycles of 94°C for 45s, 57°C for 60s, and 72°C for 90s with a final extension for 5 min. Electrophoresis and visualizing were performed under the same conditions described above.

### Sequencing

The new PCR fragments of five *L. tropica* isolates were excised from the gel and purified by Bioneer gel purification kit (Cat.No.K-3035) according to the manufacturer's instruction. The yielded products were submitted to Bioneer Company (Korea) and subjected to sequencing by Applied Biosystem automated sequencer (3730 XL). Sequences analysis result was shown through Bio Edit software version 7.0.5.3 (24).

## Results

The 27 smear samples collected from the patients in Bam district showed a 750 bp size band in agarose gel electrophoresis of kDNA amplification indicating that all were *L. tropica*, while eight out of the 13 smear preparations collected from Shiraz were identified as *L. major* (560 bp band size) and five out of 13 samples were confirmed as *L. tropica*. In addition, amastigotes observed in bone marrow aspiration obtained from the single kala-azar sample taken from a hospitalized patient were identified as *L. infantum* ( 680 bp band size).

**Fig. 1:**
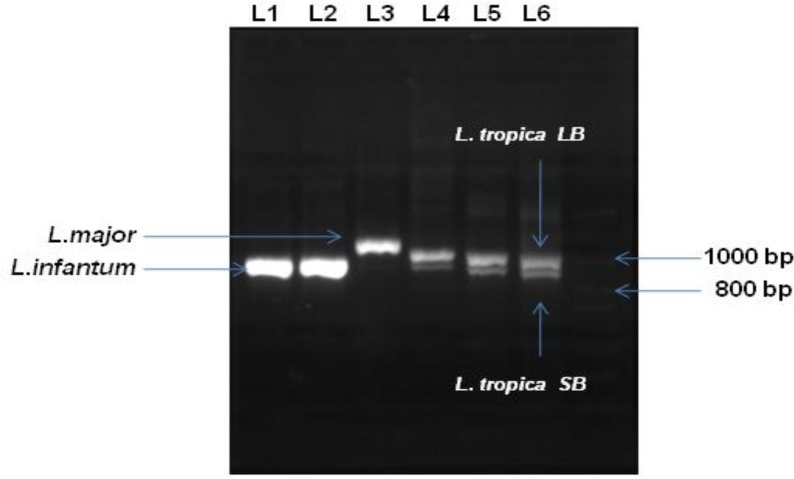
Agarose gel electrophoresis of ITS-PCR products of different species of *Leishmania*: L1 and L2 samples are related to *L. infantum* (1040 bp). L3 is a *L. major* sample (1100 bp). Both former species revealed single bands. Samples L4 to L6 showing a two-band pattern are *L. tropcia*. In case of *L. tropica*, a large size band (LB, 1050 bp) and a small size band (SB 950bp) were observed for each sample

**Fig. 2:**
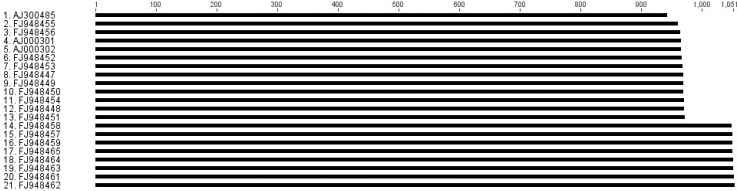
Twenty one sequences of *L. tropica* were taken from GenBank: numbers 1 to 13 (group A) have about 950 bp size and numbers 14 to 21 (group B) are sequences with 1050 bp size

Amplification of entire ITS showed a two-band pattern of about 950 bp and 1050 bp in all *L. tropica* isolates, while all *L. major* and *L. infantum* isolates had a sharp single band. *L. infantum* band size was similar to the larger band of *L. tropica*. Besides, the size of the single band of *L. major* species was about 1100 bp (Fig. 1). Analysis of ITS sequences obtained from GenBank showed sequences with sizes ranging from1110 to 1130 bp for *L. major* and with the size of 1040 bp for *L. infantum* (data not shown); however, analysis of *L. tropica* entire ITS sequences revealed two groups of sequences based on length size, the first group with the size of 950 bp and the second having fragments with the size of 1050 bp (Fig. 2).

**Fig. 3:**
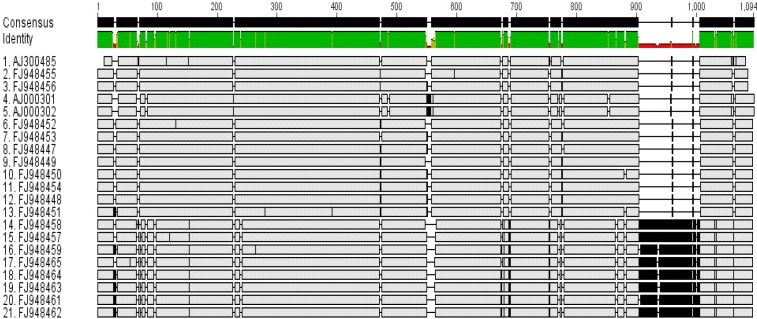
Multiple alignments of *L. tropica* sequences revealing a 100 bp gap in smaller sequences (first 13 se-quences) in comparison to larger sequences (last eight sequences). The illustration was exported from Gene-ious Basic 5.5.6 software

Multiple alignments of the GenBank sequences for three *Leishmania* species showed an interesting pattern within *L. tropica* sequences. In reality, this pattern is due to a100 bp gap within the first group (950 bp length size) in comparison to the second group (1050 bp length size) (Fig. 3).

**Fig. 4:**
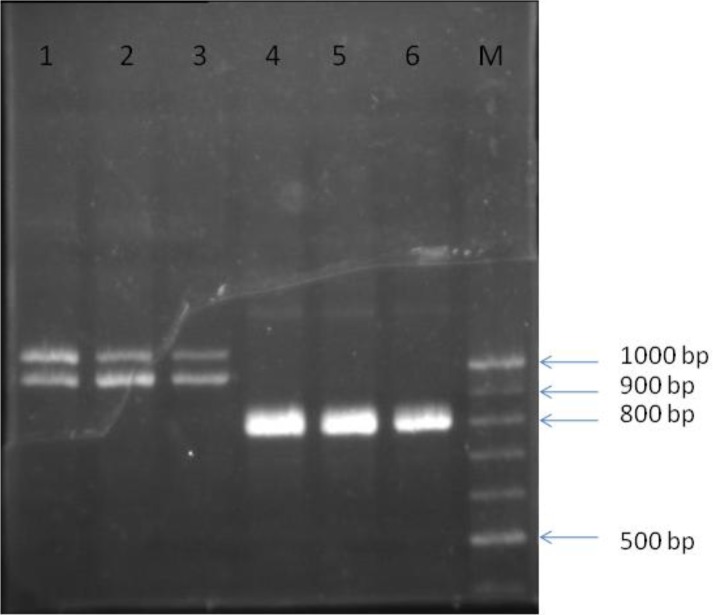
Lanes 1-3 are PCR products of whole ITS region using the former primer pair LITSR and LITSV for *L. tropica* cases from Bam district. Lanes 4-6 are same samples after amplification with the LITSR and designed LITS-MG primer. Exchange of LITSV with LITSMG reverse primer which was annealed on the site before the found gap resulted in producing single band

This pattern was observed in neither *L. infantum* nor *L. major*. Amplification of the same DNAs with the newly-designed primer, LITS-MG as the reverse primer against the forward primer LITSR, resulted in single band products (ITSMG amplicon) with about 800 bp band size (Fig. 4).

The amplicon included complete ITS1, 5.8S rRNA, and a part of ITS2 regions. It should be noted that this finding was consistent with the result obtained through *in silico* analysis. This band was obviously sharper and stronger compared to the bands resulting from electrophoresis of *L. tropica* using former primer and is suggested for further analysis, such as sequencing.

**Fig. 5:**
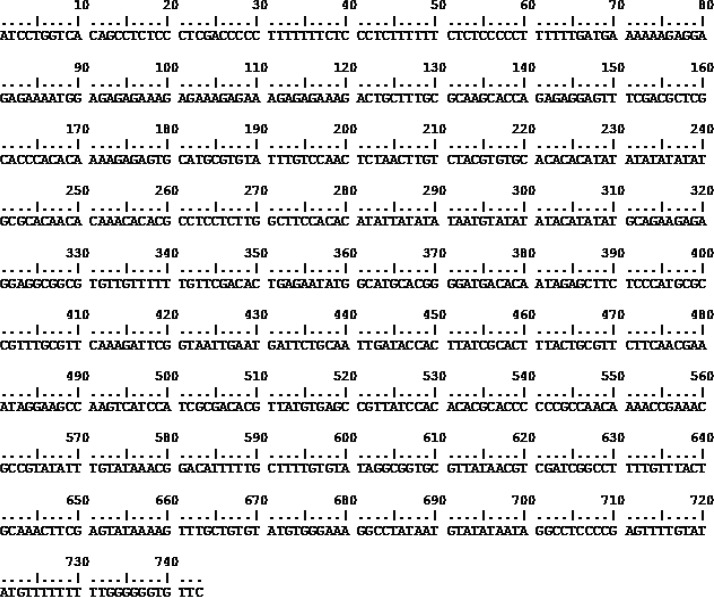
ITS MG sequence of isolates collected from Bam district, south east Iran, including 740 nucleotides after trimming both sequence tails. The sequence is shown through Bio Edit version 7.0.5.3

Multiple alignments were carried out for the sequences of *L. tropica* isolates obtained in this study from Bam region. Sequence analysis of PCR products of ITSMG fragment showed complete similarity between the five isolates in all nucleotides. Fig. 5 shows the nucleotides of representative sequence.

## Discussion

In this study, we focused on the double band electrophoretic pattern *of* *L. tropica* whole ITS-PCR products achieved by the primers LITSR and LITSV. Analysis of Gen Bank sequences of *L. tropica* ITS sequences confirmed the existence of two groups of sequences with sizes identical with bands obtained on the agarose gel. Size difference in ITS sequences is because of a 100bp gap within the sequences with shorter size. Selection of the primer (LITSMG) just before the gap resulted in expectedly single band products which reconfirmed our finding. Use of newly designed primer and amplification of ITS fragment resulted in one strong sharp band which is appropriate for following nucleotide sequencing or RFLP. Moreover, in comparison to studying ITS1 and ITS2 in apart runs, ITSMG amplicon sequences may show more variation due to covering larger fragments. This is also more economical due to the substitution of one step PCR rather than two apart PCR runs. In our study, the results of sequences analysis of ITSMG amplicon PCR products revealed the homogeneity of *L. tropica* sequences in Bam district, however, considering the low number of samples, this hypothesis should be checked in a larger analysis. This double-band pattern for whole ITS may also be applied in case of *L. tropica* in order to discriminate *L. tropica* from *L. infantum* or *L. major* in defined regions such as Bam districts..

The most important finding of study is elucidation of a 100 bp gap in *L. tropica* ITS. Result showed existence of at least two alleles for ITS in ribosomal DNA. Mauricio et al. (25) also reported at least two alleles for *L. tropica* ITS based on the total RFLP fragments size that was greater than amplification product. Schönian et al. reported a double-band pattern of ITS-rDNA for some strains of *L. tropica* as well (11). Sizes of obtained bands were similar with the sizes which observed in electrophoresis of Bam isolates. They reported that single-band ITS was observed only in some isolates. Among them small single band (960 bp) was observed for Namibian isolates, while larger single band (1060 bp) was revealed in Indian isolate and one strain with Middle East origin. Our personal communications with some Iranian researchers confirmed the existence of double-band electrophoretic pattern of *L. tropica* whole ITS for isolates obtained from some other endemic regions of Iran. Regarding Schönian and her colleagueswork in 2001(18), correlation between different geographical origin and expression of each of the three ITS special electrophoretic patterns i.e. small single-band, large single band and double-band, may be conceivable for *L. tropica*, which of course should be extensively tested using isolates from different *L. tropica* endemic region in the world.

Existence of different ITS electrophoretic patterns and difference in related alleles due to a 100 bp gap in one allele may propose probable recombination in *Leishmania*. Some evidences have strengthened hypothesis of recombination such as hybrid species of *L. major* and *L. arabica* (infecting dog and desert rat) with intermediate isoenzymatic pattern in Saudi Arabia (26, 27) or a hybrid strain found where *L. braziliensis* and *L. panamensis* cause CL in Nicaragua (28). Recombination was also shown in nagt (N-Acetyl Glucosamine-1-Phospate Transferase) enzyme in *L. tropica* (29) or hybridization between different phenotypic strains (due to isoenzymatic pattern) of *L. donovani* (MON1 and non MON1) in Tunisia (30). Another clue is the detection of a strain (LEM3946) by multilocus sequence typing which is probably a hybrid across two genetic groups (Middle East group and Sudanese group) in *L. donovani* complex (31).Significant genetic variation in some species especially *L. tropica* (32) and fusion of different species promastigotes in culture (33) or sandfly gut (34) are the other evidences which strengthen the hypothesis of recombination and sexual reproduction. Furthermore, hybridization has also been verified in *Trypanosoma*, another genus of Trypanosomatidae family (35–38). Importance of recombination and genetic exchange is on the matters of medical and taxonomic fields, especially drug resistance and pathogenicity alteration in different clones (26). Overall, although the above-mentioned explanations directs our attention to recombination hypothesis in *Leishmania*, we emphasize that our evidence is only a clue and further studies are required for elucidation and verification of genetic basis of sequence alteration responsible for this pattern of ITS.

## Conclusion

Concomitant existence of at least two alleles with a 100 bp fragment difference within ITS sequence explained the entire ITS double band electrophoretic pattern. This whole ITS double band pattern may differentiate *L. tropica* from *L. infantum* and *L. major* and can be used for confirmation of *L. tropica* species. Moreover, this evidence may empower findings of other researchers about probable recombination of *Leishmania* especially *L. tropica*. Using newly designed LITSMG primer a sharp single band was obtained which was suitable for following sequence analysis.
